# Experimental evolution of phytoplankton fatty acid thermal reaction norms

**DOI:** 10.1111/eva.12798

**Published:** 2019-04-23

**Authors:** Daniel R. O'Donnell, Zhi‐yan Du, Elena Litchman

**Affiliations:** ^1^ W. K. Kellogg Biological Station Michigan State University Hickory Corners Michigan; ^2^ Department of Integrative Biology Michigan State University East Lansing Michigan; ^3^ Program in Ecology, Evolutionary Biology and Behavior Michigan State University East Lansing Michigan; ^4^ Department of Biochemistry and Molecular Biology Michigan State University East Lansing Michigan; ^5^ MSU‐DOE Plant Research Laboratory Michigan State University East Lansing Michigan

**Keywords:** fatty acid, reaction norm, saturation, temperature, *Thalassiosira pseudonana*, thermal adaptation

## Abstract

Temperature effects on the fatty acid (FA) profiles of phytoplankton, major primary producers in the ocean, have been widely studied due to their importance as industrial feedstocks and to their indispensable role as global producers of long‐chain, polyunsaturated FA (PUFA), including omega‐3 (ω3) FA required by organisms at higher trophic levels. The latter is of global ecological concern for marine food webs, as some evidence suggests an ongoing decline in global marine‐derived ω3 FA due to both a global decline in phytoplankton abundance and to a physiological reduction in ω3 production by phytoplankton as temperatures rise. Here, we examined both short‐term (physiological) and long‐term (evolutionary) responses of FA profiles to temperature by comparing FA thermal reaction norms of the marine diatom *Thalassiosira pseudonana* after ~500 generations (ca. 2.5 years) of experimental evolution at low (16°C) and high (31°C) temperatures. We showed that thermal reaction norms for some key FA classes evolved rapidly in response to temperature selection, often in ways contrary to our predictions based on prior research. Notably, 31°C‐selected populations showed higher PUFA percentages (including ω3 FA) than 16°C‐selected populations at the highest assay temperature (31°C, above *T. pseudonana's* optimum temperature for population growth), suggesting that high‐temperature selection led to an evolved ability to sustain high PUFA production at high temperatures. Rapid evolution may therefore mitigate some of the decline in global phytoplankton‐derived ω3 FA production predicted by recent studies. Beyond its implications for marine food webs, knowledge of the effects of temperature on fatty acid profiles is of fundamental importance to our understanding of the mechanistic causes and consequences of thermal adaptation.

## 1 INTRODUCTION

Temperature affects practically every aspect of physiology, from rates of respiration (Beamish & Mookherjii, 1964; Bradford, 2013; Caron, Goldman, & Dennett, 1986), photosynthesis (Neori & Holm‐Hansen, 1982; Pastenes & Horton, 1996; Robarts & Zohary, 1987), and reproduction to (Knoblauch & Jorgensen, 1999; Lee & Ahn, 2000; Thomas, Kremer, Klausmeier, & Litchman, 2012) body size (Lindmark, Huss, Ohlberger, & Gårdmark, 2018; Montagnes & Franklin, 2001; Peter & Sommer, 2013). As an organism's temperature rises, the physical properties of cellular structures change: Proteins change conformation or denature (Corkrey et al., 2014; Ratkowsky, Olley, & Ross, 2005), and membranes become more fluid and permeable to solutes (reviewed in Hazel, 1995). These changes necessitate the evolution of strategies to maintain cellular function across a range of temperatures. Rates of resource acquisition, efficiencies of resource use (Bradford, 2013), and strategies for storing resources such as carbon, nitrogen, and phosphorus can lead to changes in relative resource requirements (Thrane, Hessen, & Andersen, 2017), internal stoichiometry (Sakamoto & Bryant, 1998; Thrane et al., 2017), and fatty acid (FA) content and composition (Canvin, 1965; James, Al‐Hinty, & Salman, 1989; Teoh, Chu, Marchant, & Phang, 2004; Thompson, Guo, Harrison, & Whyte, 1992b). Any of these traits, if measured at multiple temperatures within an organism's thermal niche, can be shown to have thermal reaction norms describing their temperature‐dependent plasticity. Evolutionary adaptation to temperature can result in changes to the slope, curvature, and offset of these reaction norms (Izem & Kingsolver, 2005; Murren et al., 2014; O'Donnell, Hamman, Johnson, Klausmeier, & Litchman, 2018).

One strategy for maintaining membrane homeoviscosity and function across temperature is to change the FA composition of the membrane (Sinensky, 1974). FA in cell walls and organelle membranes may become increasingly unsaturated at low temperatures (Jiang & Gao, 2004; Joh, Yoshida, Yoshimoto, Miyamoto, & Hatano, 1993; Patterson, 1970) as unsaturated FA pack together loosely, conferring membrane fluidity and permeability; conversely, membrane FA tends to be more saturated at high temperatures (Murata & Los, 1997; Quinn, 1981; Wada, Gombos, & Murata, 1990; Zhu, Lee, & Chao, 1997), as saturated FA (SFA) packs together in an ordered fashion, conferring membrane rigidity (Converti, Casazza, Ortiz, Perego, & Borghi, 2009; Murata & Los, 1997; Quinn, 1981).

Environmentally induced plasticity in FA profiles has been studied in a wide variety of taxa (Sinensky, 1974; reviewed in Neidleman, 1987); however, the FA profiles of phytoplankton have attracted particular attention in part due to their potential utility as feedstock for production of biofuels (Converti et al., 2009; Hu et al., 2008; Williams & Laurens, 2010) and to their global importance as a leading source of ω3 FA required by animals at higher trophic levels (Budge et al., 2014; Kainz, Arts, & Mazumder, 2004; Litzow, Bailey, Prahl, & Heintz, 2006). Published trends in algal FA profiles across temperature are extremely variable, even within a given functional group (e.g., diatoms); however, a number of generalities seem to exist. Specifically, (a) overall FA content is generally highest at the thermal niche boundaries (Patterson, 1970; Thompson, Guo, & Harrison, 1992a; Van Wagenen et al., 2012), which results in an overall negative correlation between population growth rate and FA content. (b) Percent SFA tends to increase with temperature, especially below the thermal optimum for population growth (*T*_opt_). (c) Below *T*_opt_, % polyunsaturated FA (PUFA) tends to decrease with temperature. (d) Trends in % monounsaturated FA (MUFA) across temperature are variable and often nonsignificant (Patterson, 1970; Thompson et al., 1992b; Renaud, Thinh, Lambrinidis, & Parry, 2002; Pasquet et al., 2014). Deviations from these trends (e.g., PUFA increasing and SFA decreasing with increasing temperature: Thompson et al., 1992b) can be found in many of the studies cited here.

An ecologically important consequence of reduced PUFA production at high temperatures is the potential decline in global ω3 FA production by phytoplankton as sea surface temperatures rise due to changing climate (Hixson & Arts, 2016; Kang, 2011). Phytoplankton are the primary source of ω3 FA that are essential to the survival of marine and freshwater animals at the lowest trophic levels (e.g., zooplankton: Kainz et al., 2004) to the highest (e.g., fish: Litzow et al., 2006), and to the health of the human populations that rely on the oceans for food (Budge et al., 2014; Kang, 2011). A comprehensive knowledge of the ecological and evolutionary effects of warming on ω3 FA production in phytoplankton is thus indispensable if we are to predict the effects of climate change on marine ecosystems, including the humans who depend on them.

Plasticity in FA profiles has been characterized for many phytoplankton species. However, the aim of this study was to investigate evolutionary change in temperature‐dependent reaction norms for total FA content, FA composition, and overall saturation/unsaturation of FA in response to long‐term experimental selection at low (16°C) and high (31°C) temperatures. We hypothesized three alternative scenarios for evolution of FA thermal reaction norms in phytoplankton. (a) A hot‐cold tradeoff: While an increase in temperature can lead to greater FA saturation over the short term via physiological plasticity (Murata & Los, 1997; Wada et al., 1990; Zhu et al., 1997), prolonged exposure (over many generations) to an extreme temperature may lead to constitutive production of a FA profile adapted to that temperature, that is, a reduction in plasticity. (b) Alternatively, thermal adaptation may simply lead to increased plasticity in the FA profile, contributing to (or resulting from) the broadening of the thermal niche (see Izem & Kingsolver, 2005; Kingsolver, 2009). (c) Finally, assuming certain FAs are essential components of cellular machineries beyond membranes (as may be the case for some PUFA: Sijstma & de Swaaf, 2004; Guschina & Harwood, 2006), temperature selection may bolster phytoplankton cells' ability to produce PUFA at high temperatures.

Thermal reaction norms for per capita population growth have been shown to evolve rapidly in response to experimental selection in phytoplankton (Listmann, LeRoch, Schlüter, Thomas, & Reusch, 2016; O'Donnell et al., 2018; Padfield, Yvon‐Durocher, Buckling, Jennings, & Yvon‐Durocher, 2015) and other organisms (Geerts et al., 2015; Knies, Izem, Supler, Kingsolver, & Burch, 2006; Mongold, Bennett, & Lenski, 1996). However, only a few studies have examined the ecological, metabolic, biochemical consequences of these changes (O'Donnell et al., 2018; Padfield et al., 2015; Schlüter et al., 2014). Knowledge of evolutionary changes in the temperature dependence of cellular chemistry is crucial for achieving a comprehensive understanding of the effects of temperature selection at the level of the organism. This study is among the first to examine the effects of experimental thermal adaptation on the fatty acid profile of a phytoplankton species (but see Baker et al., 2018; Pittera et al., 2018).

## 2 MATERIALS AND METHODS

### 2.1 500‐generation temperature selection experiment

The temperature‐dependent growth assays described here were conducted after ~500 generations of thermal adaptation at 16°C and 31°C (respectively, below and above the ancestral thermal optimum for per capita population growth: Boyd et al., 2013), a detailed account of which can be found in O'Donnell et al. (2018). Briefly, we obtained *Thalassiosira pseudonana* CCMP1335 (Hustedt) Hasle *et* Heimdal from the Provasoli‐Guillard National Center for Culture of Marine Phytoplankton (CCMP), Maine, USA; this strain had been maintained in semicontinuous culture at 14°C since its isolation in 1958 (Guillard & Ryther, 1962). We maintained five replicate populations (all 10 populations derived from the same single progenitor cell) in exponential growth under cool, white, fluorescent bulbs at a photon flux density of 110 µmol photons m^−2^ s^−1^ on a 14:10 light:dark cycle, transferring 10^6^ cells into fresh L1 marine culture medium (Guillard & Hargraves, 1993; modified by O'Donnell et al., 2018) every 4 days, with occasional deviations of ±1 day. After the selection experiment, all populations were cryopreserved in liquid nitrogen until needed, at which point they were revived for assays.

### 2.2 Cryopreservation and revival

To cryopreserve *T. pseudonana* populations, we first grew them to late log‐phase in their selection (“home”) temperature environments before placing 1 ml of culture in a 2‐ml cryogenic vial. We then added 1 ml of 24% dimethyl sulfoxide (76% L1 medium), for a final DMSO concentration of 12%. After 20 min, cryogenic vials were placed in a passive freezer unit (Mr. Frosty, Thermo Scientific, USA) containing 250 ml isopropyl alcohol, and cooled to −80°C at ~1°C/min, after which vials were plunged and stored in liquid nitrogen (−196°C).

We revived cultures 2 weeks prior to beginning acclimation for temperature‐dependent assays. Under dark conditions, we thawed cryogenic vials by floating them in 500‐ml beakers full of 20°C tap water. We immediately emptied each thawed culture into a 20‐ml scintillation vial containing 10 ml of L1 medium, also at 20°C. Scintillation vials were loosely capped and placed in a light‐proofed cardboard box at 20°C for 36 hr (as in Hipkin, Day, Rad‐Menéndez, & Mock, 2013), after which they were placed under the light conditions described above. Each vial was gently inverted daily to keep cells in suspension. As soon as a revived culture grew to a visible density, we transferred it to a 50‐ml tissue culture flask containing 20 ml of L1 medium. Starting at this point, all cultures were maintained at 20°C for 10 generations prior to acclimation at assay temperatures.

Cryopreservation of diatoms is a well‐documented practice (McClellan, 1989; Cañavate & Lubián, 1997; Buhmann, Day, & Kroth, 2013; Hipkin et al., 2013; Stock et al., 2018. *Thalassiosira pseudonana* has been shown to have a survival rate of up to 75% and to be genotypically stable during cryopreservation using a method similar to ours (Hipkin et al., 2013). We acknowledge that cryopreservation may result in altered allele frequencies and potential loss of rare alleles in some cases; however, significant loss of diversity seems unlikely (Sprouffske, Aguilar‐Rodríguez, & Wagner, 2016).

### 2.3 Algal growth and harvesting

We assayed all five replicate *T. pseudonana* populations from each experimental selection temperature group (16°C and 31°C) in triplicate at 10, 16, 26, and 31°C (2 selection temperatures × 4 assay temperatures × 5 populations × 3 replicates = 120 total cultures). We chose these temperatures to achieve broad coverage of *T. pseudonana*'s physiology at temperatures spanning the majority of its thermal tolerance range and to include the two selection temperatures and one temperature (26°C) approximating *T. pseudonana'*s optimal temperature for population growth (Boyd et al., 2013; O'Donnell et al., 2018). We conducted acclimations and assays in temperature‐controlled growth chambers, under the light conditions described above. Each replicate population was maintained in exponential growth for a 10‐generation acclimation period at each assay temperature prior to the assay to account for possible trans‐generational, nongenetic effects (Kremer, Fey, Arellano, & Vasseur, 2018). From each replicate population, we transferred 10^5^ cells to each of three 50‐ml polystyrene tissue culture flasks containing 40 ml of sterile‐filtered L1 medium. We monitored relative change in cell densities daily by placing each tissue culture flask in a spectrophotometer (Shimadzu UV‐2401PC; Shimadzu Corporation) and measuring Abs_436_, corresponding to the absorbance peak of chlorophyll *a* (Neori, Vernet, Holm‐Hansen, & Haxo, 1986).

We harvested all 120 cultures in late log‐phase; time to harvest varied by temperature from 4 days at 26°C to nearly 2 weeks at 10°C. We filtered 15 ml of culture through a Whatman GF/B glass fiber filter for fatty acid analysis and froze these samples at −20°C. We retained 25 µl for population density estimates, obtained using a CASY particle counter (Schärfe System GmbH, Reutlingen, Germany). The remaining ~25 ml of each culture was filtered for analyses not addressed here.

### 2.4 Fatty Acid Methyl Ester reaction and Gas Chromatography

To analyze *T. pseudonana* fatty acid profiles, we performed fatty acid methyl ester (FAME) reactions on each GF/B filter sample according to the protocol by Wang and Benning (2011) and adapted for algae by Boyle et al. (2012). We first extracted lipids from the algal samples described above using a solution of chloroform, methanol, and formic acid (10:20:1). After washing with a buffer (0.2M H_3_PO_4_ + 1 M KCl), we spun samples down for 3 min at 2000 *g*, transferred the remaining chloroform layer to a fresh tube, and dried the samples with a stream of N_2_ gas. After drying, we added 100 µl of internal 15:0 standard (50 µg/ml pentadecanoic acid), then proceeded with the FAME protocol (Boyle et al., 2012; Wang & Benning, 2011), and quantified fatty acids using an Agilent Technologies 7890A Gas Chromatography system (Agilent Technologies). We used the Agilent capillary DB‐23 column for FAME analysis, with the following temperature settings: initial temperature 140°C, increased by 30°C/min to 160°C, then by 8°C/min to 240°C, and held at 240°C for 2 min. Samples in which the GC failed to detect ≥50% of the FA classes were dropped from statistical analyses.

### 2.5 Calculations

All percentages presented here are calculated based on molar masses of fatty acid molecules on a per‐biovolume basis (µ/m^3^). The cell volumes used for this calculation are mean (*n* = 10) cylindrical volumes (πr^2^h) calculated from length and width measurements of *T. pseudonana* cells in girdle view, under 1000× magnification (oil immersion; see data at Dryad link below). Individual fatty acids are presented as percentages of total fatty acid weight (molar). Mean chain length (*MCL*) is calculated as $MCL=\sum_{i=1}^{16}\frac{mol\;FA_{i}}{mol\;FA_{\mathrm{tot}}}\times lc_{i},$ where *mol FA_i_* is the weight in moles of a given fatty acid, *mol FA_tot_* is the total weight of all fatty acids, and *lc_i_* is the length (number of carbon atoms) of *FA_i_* (Renaud et al., 2002). Degree of unsaturation is calculated as $WUnSat=\sum_{i=1}^{16}\frac{mol\;FA_{i}}{mol\;FA_{\mathrm{tot}}}\times ndb_{i},$ where the first term is as in *MCL*, and *ndb_i_* is the number of double bonds in *FA_i_* (Thompson et al., 1992b). Percent ω3 FA is the sum of %16:3, %16:4, %18:3, %20:5 (eicosapentaenoic acid, EPA), and %22:6 (docosapentaenoic acid, DHA) FA.

### 2.6 Linear mixed‐effects models

The same mixed model, fit using the “lme” function from R package “nlme” (Pinheiro, Bates, & R Core Team, 2017), was used to test effects of *selection temperature*,* assay temperature*, and a *selection temperature* × *assay temperature* interaction on total fatty acid (FA) production, %SFA, %MUFA, %PUFA, mean chain length (MCL), unsaturation (WUnSat), and percentages of individual FA classes and of %ω3 FA. We included *selection line* as a random effect to account for within‐replicate repeated measures across assay temperatures; we also included a random effect at the level of the *assay replicate* (note that assays were run in triplicate), to account for duplicated FAME analyses conducted on each of the triplicates. Finally, we included two variance functions (allowing for different variances by factor level: Pinheiro et al., 2017) to account for unequal variances between selection temperature levels and among assay temperature levels. These variance functions are included as weights in the model using the “varIdent” function from nlme (Pinheiro et al., 2017).

## 3 RESULTS

**Figure 1:**
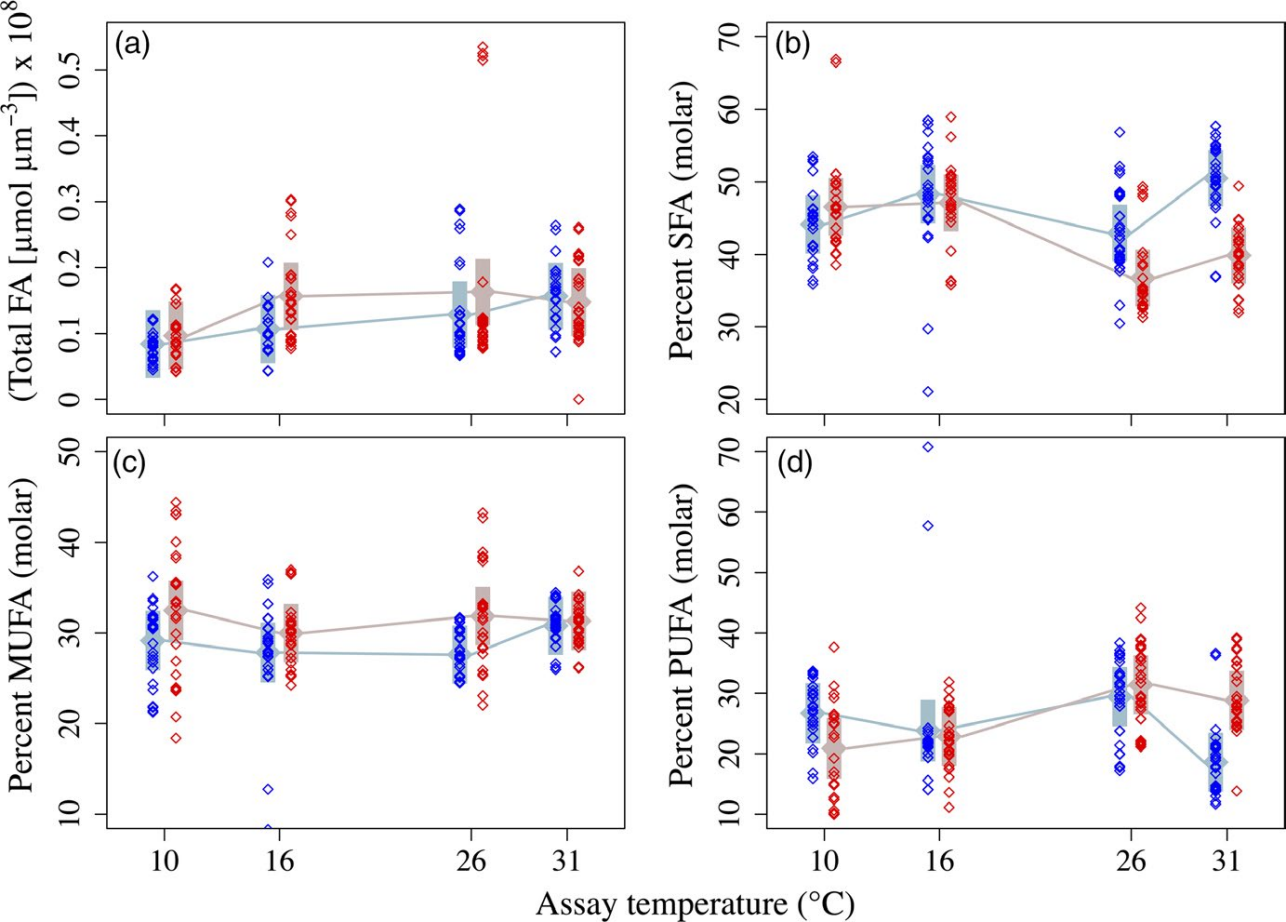
(a) Total fatty acid (FA) content per biovolume (µmol/µm^3^) in 16°C‐selected (blue) and 31°C‐selected (red) populations at 10, 16, 26, and 31°C. Per‐biovolume FA content was estimated by dividing the total FA content of the sample by the population density and then by the mean cell volume (*n* = 10 cells). Note that the y‐axis is multiplied by 10^8^ for clarity. (b) Saturated FA as a fraction of total FA; (c) monounsaturated FA as a fraction of total FA; (d) polyunsaturated FA as a fraction of total FA. *n* = 3 for each replicate population. Grayish symbols represent fitted means (fixed effects) from linear mixed‐effects models, with 95% confidence intervals

As in other marine diatoms (Harwood & Jones, 1989), *T. pseudonana* contains FA ranging in length from C_14_ to C_22_, with substantial representation of the long‐chain, essential ω3 fatty acids EPA and DHA (C_20_ and C_22,_ respectively). The dominant fatty acid in both 16°C‐ and 31°C‐selected populations was 16:1^Δ3^ at all four assay temperatures (for molar FA profiles by selection and assay group, see Supplemental Information Figure S1). Total fatty acid content per biovolume (µm^3^) trended higher in 31°C‐selected populations than in 16°C‐selected populations, though not significantly (Table 1; Figure 1a), and total fatty acid content was lowest in both groups at 10°C and highest at 31°C (Table 1; Figure 1a). The percentage of total FA represented by saturated fatty acids (SFAs) was higher in 16°C‐selected populations than in 31°C‐selected populations, though not at the 10°C assay temperature; the difference in %SFA was especially pronounced at the 31°C assay temperature, where %SFA in 31°C‐selected populations was ~20% lower than in 16°C‐selected populations (Table 1; Figure 1b). Percent monounsaturated FA (%MUFA) was ~10% higher in 31°C‐selected populations than in 16°C‐selected populations, but did not vary across assay temperatures (Table 1; Figure 1c). Percent polyunsaturated FA (%PUFA) was not different between the two selection groups at 10, 16, and 26°C, however 16°C‐selected populations experienced a ~14% decrease in %PUFA between 26°C and 31°C, leading to a significant difference in %PUFA between the two groups at the 31°C assay temperature (Table 1; Figure 1d).

**Table 1:** RM‐ANOVA testing effects of selection temperature and assay temperature on *Thalassiosira pseudonana* mean chain length (*MCL*), unsaturation (*WUnSat*), total fatty acids (total FA), percent saturated FA (%SFA), percent monounsaturated FA (%MUFA), percent polyunsaturated FA (%PUFA)

Trait	RM test	*df*	*F*‐statistic	*p*‐value
*MCL*	Selection temp.	1,8	1.92	0.20
	Assay temp.	3,101	37.48	<0.0001
	Assay temp. × Sel. Temp.	3,101	3.36	0.086
*WUnSat*	Selection temp.	1,8	0.79	0.30
	Assay temp.	3,101	10.04	<0.0001
	Assay temp. × Sel. Temp.	3,101	6.47	0.0005
*Total FA (mol)*	Selection temp.	1,8	1.23	0.40
	Assay temp.	3,101	5.00	0.0028
	Assay temp. × Sel. Temp.	3,101	1.07	0.37
*%SFA*	Selection temp.	1,8	13.67	0.0061
	Assay temp.	3,101	10.04	<0.0001
	Assay temp. × Sel. Temp.	3,101	7.38	0.0002
*%MUFA*	Selection temp.	1,8	5.20	0.052
	Assay temp.	3,101	1.52	0.21
	Assay temp. × Sel. Temp.	3,101	1.04	0.38
*%PUFA*	Selection temp.	1,8	0.91	0.37
	Assay temp.	3,101	7.38	0.0002
	Assay temp. × Sel. Temp.	3,101	7.06	0.0002
*%ω3 fatty acids*	Selection temp.	1,8	0.23	0.64
	Assay temp.	3,101	9.89	<0.0001
	Assay temp. × Sel. Temp.	3,101	4.31	0.0066

**Figure 2:**
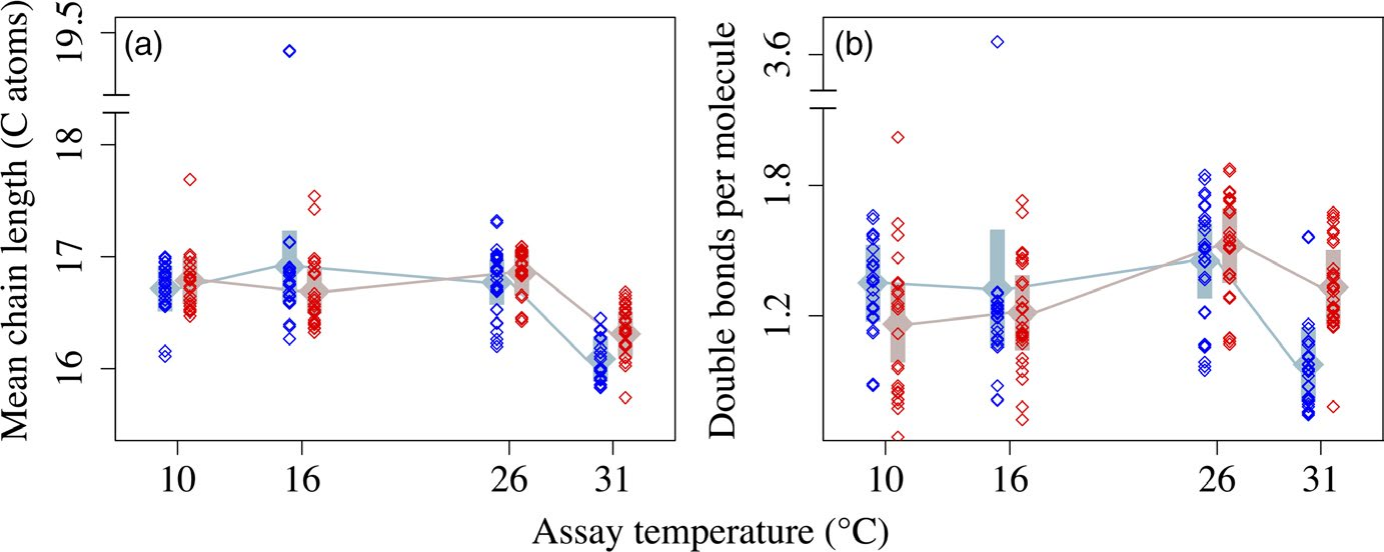
(a) Mean chain length (C atoms per molecule) and (b) degree of unsaturation (double bonds per molecule) in 16°C‐selected (blue) versus 31°C‐selected (red) *Thalassiosira pseudonana* populations at 10, 16, 26, and 31°C. Mean chain length was estimated as $MCL=\sum_{i=1}^{16}\frac{mol\;FA_{i}}{mol\;FA_{\mathrm{tot}}}\times lc_{i},$ where *mol FA_i_* is the weight in moles of a given fatty acid, *mol FA_tot_* is the total weight of all fatty acids, and *lc_i_* is the length (number of carbon atoms) of *FA_i_*. Unsaturation was estimated as $WUnSat=\sum_{i=1}^{16}\frac{mol\;FA_{i}}{mol\;FA_{\mathrm{tot}}}\times ndb_{i},$ where the first term is as in MCL, and *ndb_i_* is the number of double bonds in *FA_i_*. *n* = 3 for each replicate population. Gray symbols represent fitted means (fixed effects) from linear mixed‐effects models, with 95% confidence intervals. Note the axis breaks in both panels

**Figure 3:**
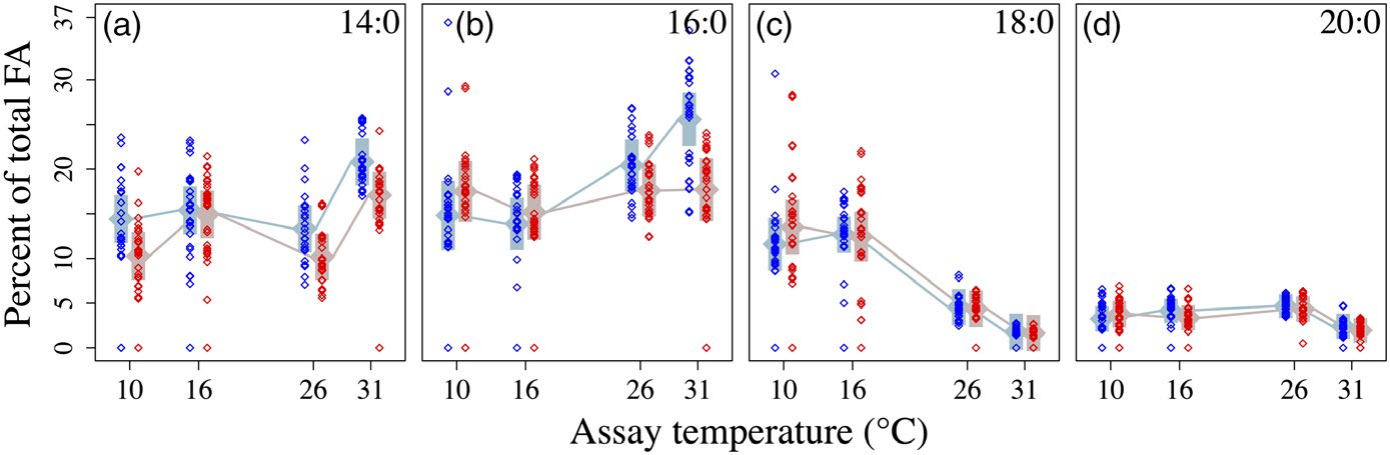
Saturated fatty acids as percentages of total fatty acids in 16°C‐selected (blue) versus 31°C‐selected (red) *Thalassiosira pseudonana* populations at 10, 16, 26, and 31°C. *n* = 3 for each replicate population. Numbers in the upper right corners indicate the fatty acid classes shown in each panel; for example, “14:0” is a FA with 14 C atoms and no double bonds. Other symbols are as in previous figures

**Figure 4:**
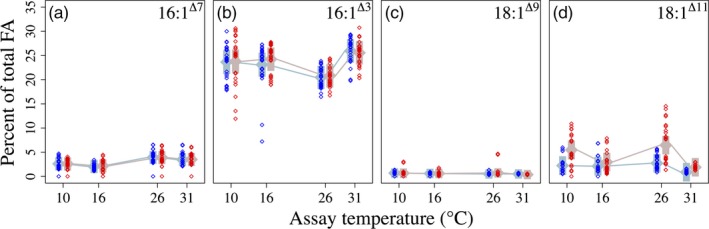
Monounsaturated fatty acids as percentages of total fatty acids in 16°C‐selected (blue) versus 31°C‐selected (red) *Thalassiosira pseudonana* populations at 10, 16, 26, and 31°C. *n* = 3 for each replicate population. Numbers in upper right corners indicate the fatty acid classes shown in each panel. “∆” indicates the position, from the carboxyl end, of the double bond; for example, “∆3” indicates a double bond in the third position from the carboxyl end. Other symbols are as above

**Figure 5:**
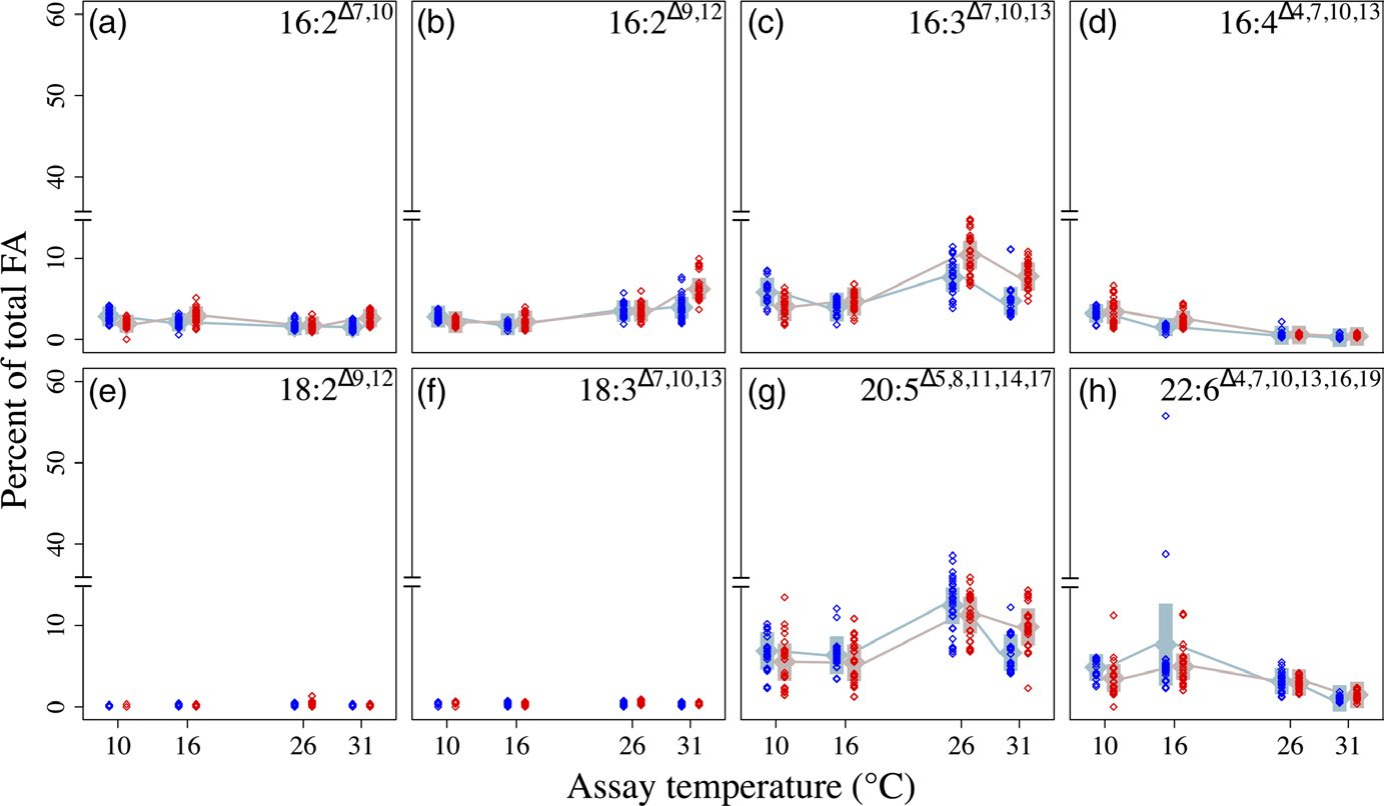
Polyunsaturated fatty acids as percentages of total fatty acids in 16°C‐selected (blue) versus 31°C‐selected (red) *Thalassiosira pseudonana* populations at 10, 16, 26, and 31°C. *n* = 3 for each replicate population. Symbols are as in previous figures. Note the axis breaks in all panels

The composite metric of FA unsaturation (*WUnSat*) was largely driven by %PUFA. Unsaturation was higher overall in 31°C‐selected populations (Table 1; Figure 2a), with the greatest difference in unsaturation at the 31°C assay temperature (Table 1; Figure 2a). There was no main effect of selection temperature on mean chain length (Table 1; Figure 2b). While informative, these composite metrics obscure the nuances in the divergence (or lack thereof) of thermal reaction norms for individual fatty acid classes. We will highlight a few notable FA classes in the text; for all others, refer to Tables S1–S3 and Figures 3, 4, 5.

Among the SFA, 16:0 FA was notable in that, at the 10°C assay temperature, 31°‐selected populations had higher %16:0, while at 26 and 31°C, 31°C‐selected populations had the highest %16:0 (Figure 3b). Percent 18:0 FA decreased ~60% between the 16°C and 26°C assay temperatures in both selection groups, dropping an additional ~25% between 26°C and 31°C (Figure 3c).

Among the MUFA, 16:1^Δ7^ and 16:1^Δ3^ FA represented between 20% and 30% of total FA in both selection groups and at all assay temperatures. However, these two FA classes differed the least between the two temperature selection groups. Thermal reaction norms for both 16:1 FA classes were parallel and differed by ≪1% at every assay temperature (Figure 4a,b). Percent 18:1^Δ11^ was ~3× higher in 31°C‐selected populations than in 16°C‐selected populations across assay temperatures (Figure 4d). Percent 18:1^Δ11^ was lowest for both selection groups at the 31°C assay temperature (*assay temperature* × *selection temperature* interaction Table S1; Figure 4d).

**Figure 6:**
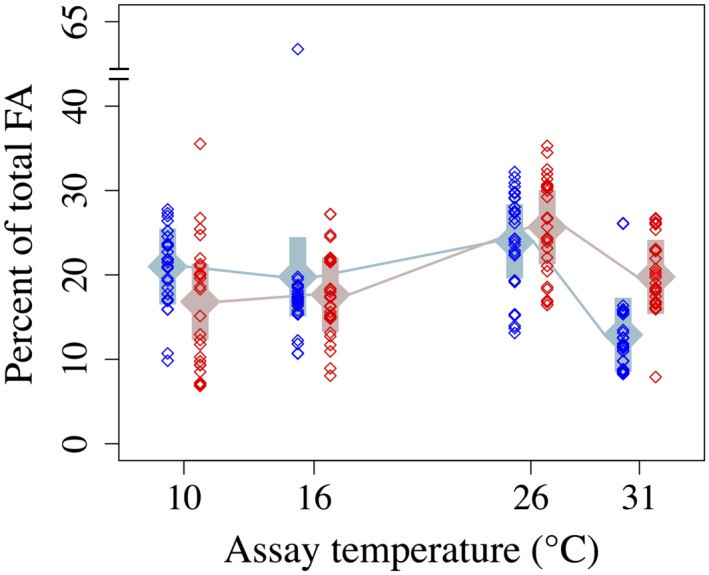
ω3 fatty acids as percentages of total fatty acids in 16°C‐selected (blue) versus 31°C‐selected (red) *Thalassiosira pseudonana* populations at 10, 16, 26, and 31°C. *n* = 3 for each replicate population. Symbols are as in previous figures. Note the axis break

Percent 16:3^Δ7,10,13^ (16:3ω3 hereafter) FA was higher in 16°C‐selected populations than in 31°C‐selected populations at 10°C, while the reverse was true at 16, 26, and 31°C (Figure 5c). Percent 16:4^Δ4,7,10,13^ (16:4ω3) FA was consistently higher in 31°C‐selected populations than in 16°C‐selected populations (Figure 5d), but decreased by roughly an order of magnitude between 10°C and 31°C assay temperatures in both selection groups (Figure 5d). 18:2^Δ9,12^ FA and 18:3^Δ9,12,15^ (18:3ω3) FA were nearly absent. Combined, they rarely exceeded 1% of total FA and were often undetectable, preventing statistical analysis. The ω3 FA 20:5^Δ5,8,11,14,17^ (EPA) and 22:6^Δ4,7,10,13,16,19^ (DHA) were the two longest (most C atoms) FA classes, and each represented between 1% and 10% of total FA. Percent EPA and %DHA did not differ between selection groups overall, but %EPA was higher in 31°C‐selected populations at the 31°C assay temperature (*assay temperature* × *selection temperature* interaction: Table S1; Figure 5g). Percent EPA was highest overall for both selection groups at 26°C (Figure 5g), while %DHA peaked for both groups at 16°C, and then declined by nearly an order of magnitude between 16°C and 31°C (Figure 5h). Notably, selection at 31°C resulted in ω3 FA making up nearly 2× the percentage of total FA at the 31°C assay temperature than selection at 16°C (Figure 6).

## 4 DISCUSSION

We showed that the temperature dependence of fatty acid profiles in *Thalassiosira pseudonana* can evolve rapidly in response to experimental temperature selection. Replicate *T. pseudonana* populations selected at low (16°C) versus high (31°C) temperatures exhibited marked divergence in FA saturation at the four assay temperatures (10, 16, 26, and 31°C), driven by major divergence in thermal reaction norms of a few key FA classes. However, the temperature‐dependent responses of fatty acid content and composition in both *T. pseudonana* temperature selection groups were idiosyncratic and often opposite predictions based on prior research (Jiang & Gao, 2004; Patterson, 1970; Sinensky, 1974; Wada et al., 1990). For example, SFAs were often higher at low temperatures and UFA higher at high temperatures.

Total FA per biovolume varied little across assay temperatures, but was lowest at 10°C and highest at 31°C for both selection groups, on average. In contrast, Patterson (1970) showed that FA as a percentage of dry weight in the chlorophyte *Chlorella sorokiniana* increased toward the thermal niche boundaries and was lowest near *T*_opt_. Van Wagenen et al. (2012) showed a similar pattern for the ochrophyte *Nannochloropsis salina*. In eight species of phytoplankton including *T. pseudonana,* Thompson et al. (1992a) found thermal reaction norms for lipid per cell to be highly variable, ranging from concave up to concave down between 10 and 25°C; of particular note, however, the reaction norm for *T. pseudonana* was distinctly concave‐up.

Percent SFA decreased slightly between 10 and 31°C in 16°C‐selected populations, with a large drop between 16 and 26°C; %SFA decreased dramatically in 31°C‐selected populations between 10°C and 31°C; and %SFA was significantly higher in 16°C‐selected populations than in 31°C‐selected populations at all assay temperatures except 10°C. These trends are unexpected (see hypothesis 1 in Introduction) but not unprecedented. Teoh et al. (2004) observed increased unsaturation at low temperatures in only one of six phytoplankton species studied. There were some indications, however, that *T. pseudonana* in our study adjusted percentages of certain PUFA as a means of maintaining homeoviscosity across temperature and that thermal reaction norms for these PUFA evolved in response to temperature selection in the direction predicted. First, %PUFA and, consequently, *WUnSat* (average double bonds per FA molecule) were slightly higher in 16°C‐selected populations than in 31°C‐selected populations at 10°C. Second, EPA, which ranged roughly from 5% to 15% of total FA in both 16 and 31°C‐selected populations, did not differ between selection groups at 10, 16, and 26°C, but was lower in 16°C‐selected populations at 31°C. It would appear, therefore, that in addition to evolving enhanced production of these highly unsaturated FA (HUFA), 16°C‐selected populations were also more plastic in their ability to regulate the amount of HUFA produced, depending on temperature. In contrast to previous studies (Jiang & Gao, 2004; Pasquet et al., 2014), %EPA did not decrease overall between 10 and 31°C, while %DHA decreased with increasing assay temperature in both groups.

Besides unsaturation, FA chain length (C atoms per molecule) has been suggested to play a role in regulating membrane fluidity (Hochachka & Somero, 1984; Sinensky, 1974). These authors suggest that microbes tend to incorporate longer‐chain FA into their membranes at high temperatures, as shorter FA molecules (especially SFA) “melt” at lower temperatures (Hochachka & Somero, 1984; Sinensky, 1974). Incorporation of short‐chain SFA (14:0 and 16:0 FA, in this case) into membranes when cold and long‐chain SFA (18:0 and 20:0 here) when warm may thus be considered a strategy for maintaining homeoviscosity across temperature. However, 14:0 FA and 16:0 FA have melting points of 53.9 and 63.1°C, respectively (Hochachka & Somero, 1984), both of which lie well outside the thermal niche of *T. pseudonana*. No SFAs detected in our study were thus ever in liquid phase. The longest‐chain SFAs in our study were 18:0 and 20:0, both of which decreased their % abundances dramatically between 10 and 31°C. Mean chain length (*MCL*), which incorporates both SFA and UFA, was fairly constant between 10 and 26°C in both *T. pseudonana* temperature selection groups and dropped significantly from 26 to 31°C. *MCL* was also marginally higher in 31°C‐selected populations than in 16°C‐selected populations at the 31°C assay temperature. While %14:0 FA was higher in 16°C‐selected populations than in 31°C‐selected populations across assay temperatures, both 14:0 FA and 16:0 FA increased by ~50% in 16°C‐selected populations between 10 and 31°C, indicating a tendency for cold‐adapted *T. pseudonana* to increase production of short, saturated FA at high, rather than at low temperatures. This is consistent with some studies more recent than Sinensky (1974) and Hochachka and Somero (1984), which have shown nonsignificant (Renaud et al., 2002; Renaud, Zhou, Parry, Thinh, & Woo, 1995; Thompson et al., 1992b) or negative (Renaud et al., 2002, 1995) trends in mean chain length with increasing temperature. In sum, favoring longer *MCL* at high temperature (and shorter at low) does not appear to be a strategy for maintaining homeoviscosity across temperature in *T. pseudonana* populations in either of our temperature selection groups, nor does this strategy appear to be the rule in other phytoplankton species.

Some well‐targeted follow‐up research could shed a great deal of light on temperature dependence of FA saturation and desaturation in temperature‐selected populations of *T. pseudonana* and in phytoplankton in general. For example, a study of mechanisms of FA desaturation at the cellular level in temperature‐selected *T. pseudonana* populations combined with information on underlying genetic change would be a powerful and illuminating follow‐up to this study. Tonon et al. (2005) identified seven desaturases in *T. pseudonana* using the draft genome by Armbrust et al. (2004). Assays of the activities of these desaturases in cold‐ and warm‐adapted *T. pseudonana* populations across a broad temperature range could be combined with the amino acid sequences of the desaturases themselves to better understand the role of FA profile evolution in *T. pseudonana*'s thermal adaptation.

Phytoplankton, especially diatoms, are a vital source of fatty acids for marine organisms at higher trophic levels and are the primary source of long‐chain, highly unsaturated FA (HUFA) in the world's marine (Budge et al., 2014; Hixson & Arts, 2016; Kang, 2011) and freshwater (Kainz et al., 2004) food webs. Global production of HUFA by marine phytoplankton may be declining as a result of rising sea surface temperatures (Hixson & Arts, 2016); climate effects on HUFA production in phytoplankton must therefore be considered of global ecological interest. We showed that, while ω3 FA as a percentage of total FA indeed decreased between 26°C and 31°C in both *T. pseudonana* selection groups, %ω3 was nearly 2× higher in the 31°C selection group than in the 16°C selection group at 31°C. This result is notable, as it suggests that adaptation to warming oceans may mitigate effects on global phytoplankton ω3 production, at least insofar as such effects are driven by physiological, rather than population responses.

Studies of the effects of warming on the content and composition of FA in phytoplankton communities have primarily focused on multispecies responses (Hixson & Arts, 2016) or shifts in phytoplankton assemblages leading to changes in the availability of vital HUFA to higher trophic levels (Budge et al., 2014; Litzow et al., 2006). Here, we showed that FA content and composition within a species of marine diatom, *Thalassiosira pseudonana*, are sensitive to temperature on both short (physiological) and long (evolutionary) timescales, and that thermal reaction norms of key FA are evolutionarily labile. Importantly, *T. pseudonana* populations experimentally adapted to 16°C and 31°C exhibited different responses to high temperature by two major ω3 FA, 16:3ω3, 20:5ω3. 16:4 and 22:6 FA declined sharply between 10 and 31°C in all experimental populations, but 31°C‐selected populations were significantly higher in combined ω3 FA at the highest assay temperature, 31°C. Thus, while warming may lead to a decrease in production of some key PUFA, *T. pseudonana* may be able to recover some PUFA production through rapid evolutionary responses to warming seas. Long‐term implications of evolutionary adaptation to warming in phytoplankton for availability of ω3 FA to higher trophic levels, including humans, are worthy of further investigation.

## CONFLICT OF INTEREST

None declared.

## Data Availability

The data underlying the main results of this study are archived on the Dryad Digital Repository: https://doi.org/10.5061/dryad.pn04m84 (O'Donnell, Du, & Litchman, 2019).
