# Supplementary material for: Experimental evolution of phytoplankton fatty acid thermal reaction norms
Source: Evol Appl. 2019 Apr 23;12(6):1201–11. doi: 10.1111/eva.12798 (PMC6866708; doi:10.1111/eva.12798)
Supplement: Supplementary file 1 [file EVA-12-1201-s001.docx]

**Supplemental Information**

Experimental evolution of phytoplankton fatty acid thermal reaction norms

Daniel R. O’Donnell; Zhi-Yan Du; Elena Litchman

**Correspondence to:** [odonnell.380@osu.edu](mailto:odonnell.380@osu.edu)

**This file includes:**

Figure S1: Fatty acid profiles of 16°C- and 31°C- selected *Thalassiosira pseudonana* populations assayed at 10, 16, 26 and 31°C.

Tables S1-S3: Repeated-measures ANOVA tables for 16 individual fatty acid classes in 16°C- and 31°C-selected *Thalassiosira pseudonana* populations assayed at 10, 16, 26 and 31°C.

**Figure S1.** Total content of 16 fatty acid classes in *T. pseudonana* evolved at 16°C (blue) and 31°C (red) and grown at 10°C, 16°C, 26°C, 31°C. In each panel, replicate selection lines are in order (1-5) from left to right for each selection group. Lines across boxes indicate the median of each group, with the lower and upper bounds indicating the 25^th^ and 75^th^ quartiles, respectively. Points beyond the lower and upper whiskers are outliers, falling outside the 0^th^ and 100^th^ quartiles. Y-axis values are multiplied by 10^10^ for clarity.

**Table S1** Repeated-measures ANOVA comparing individual saturated fatty acids (SFA) between temperature selection groups (31°C versus 16°C) and among assay temperatures (10, 16, 26 and 31°C).

| **SFA** | **Effect** | **df** | **F-statistic** | **P-value** |
| --- | --- | --- | --- | --- |
| *%14:0* | Selection temp. | 1,8 | 12.21 | 0.0081 |
|  | Assay temp. | 3,101 | 26.87 | <0.0001 |
|  | Sel. temp. × Assay temp. | 3,101 | 1.64 | 0.18 |
| *%16:0* | Selection temp. | 1,8 | 1.49 | 0.26 |
|  | Assay temp. | 3,101 | 26.93 | <0.0001 |
|  | Sel. temp. × Assay temp. | 3,101 | 14.72 | <0.0001 |
| *%18:0* | Selection temp. | 1,8 | 0.17 | 0.69 |
|  | Assay temp. | 3,101 | 144.24 | <0.0001 |
|  | Sel. temp. × Assay temp. | 3,101 | 1.90 | 0.13 |
| *%20:0* | Selection temp. | 1,8 | 1.65 | 1.24 |
|  | Assay temp. | 3,101 | 25.70 | <0.0001 |
|  | Sel. temp. × Assay temp. | 3,101 | 1.70 | 0.17 |

**Table S2** Repeated-measures ANOVA comparing individual monounsaturated fatty acids (MUFA) between temperature selection groups (31°C versus 16°C) and among assay temperatures (10, 16, 26 and 31°C).

| **MUFA** | **Effect** | **df** | **F-statistic** | **P-value** |
| --- | --- | --- | --- | --- |
| *%16:1^Δ7^* | Selection temp. | 1,8 | 0.018 | 0.96 |
|  | Assay temp. | 3,101 | 12.91 | <0.0001 |
|  | Assay temp × Sel. temp. | 3,101 | 0.033 | 0.99 |
| *%16:1^Δ3^* | Selection temp. | 1,8 | 0.16 | 0.70 |
|  | Assay temp. | 3,101 | 13.24 | <0.0001 |
|  | Sel. temp. × Assay Temp. | 3,101 | 0.30 | 0.83 |
| *%18:1^Δ9^* | Selection temp. | 1,8 | 0.023 | 0.88 |
|  | Assay temp. | 3,101 | 1.17 | 0.32 |
|  | Sel. temp. × Assay Temp. | 3,101 | 0.62 | 0.61 |
| *%18:1^Δ11^* | Selection temp. | 1,8 | 15.29 | 0.0045 |
|  | Assay temp. | 3,101 | 13.47 | <0.0001 |
|  | Sel. temp. × Assay Temp. | 3,101 | 3.66 | 0.015 |

**Table S3** Repeated-measures ANOVA comparing individual polyunsaturated fatty acids (PUFA) between temperature selection groups (31°C versus 16°C) and among assay temperatures (10, 16, 26 and 31°C).

| **PUFA** | **Effect** | **df** | **F-statistic** | **P-value** |
| --- | --- | --- | --- | --- |
| *%16:2^Δ7,10^* | Selection temp. | 1,8 | 10.21 | 0.013 |
|  | Assay temp. | 3,101 | 9.92 | <0.0001 |
|  | Sel. temp. × Assay Temp. | 3,101 | 15.88 | <0.0001 |
| *%16:2^Δ9,12^* | Selection temp. | 1,8 | 9.09 | 0.017 |
|  | Assay temp. | 3,101 | 61.02 | <0.0001 |
|  | Sel. temp. × Assay Temp. | 3,101 | 13.49 | <0.0001 |
| *%16:3^Δ7,10,13^* | Selection temp. | 1,8 | 9.38 | 0.016 |
|  | Assay temp. | 3,101 | 46.33 | <0.0001 |
|  | Sel. temp. × Assay Temp. | 3,101 | 13.03 | <0.0001 |
| *%16:4^Δ4,7,10,13^* | Selection temp. | 1,8 | 3.84 | 0.086 |
|  | Assay temp. | 3,101 | 230.90 | <0.0001 |
|  | Sel. temp. × Assay Temp. | 3,101 | 5.83 | 0.0010 |
| ** %18:2^Δ9,12^* | -- | -- | -- | -- |
| ** %18:3^Δ9,12,15^* | -- | -- | -- | -- |
| *%20:5^Δ5,8,11,14,17^* | Selection temp. | 1,8 | 0.0035 | 0.95 |
|  | Assay temp. | 3,101 | 29.74 | <0.0001 |
|  | Sel. temp. × Assay Temp. | 3,101 | 4.65 | 0.0043 |
| *%22:6^Δ4,7,10,13,16,19^* | Selection temp. | 1,8 | 0.56 | 0.48 |
|  | Assay temp. | 3,101 | 43.74 | <0.0001 |
|  | Sel. temp. × Assay Temp. | 3,101 | 2.76 | 0.046 |

* No model fit, due to zero inflation.
